# Drought-Stress Induced Physiological and Molecular Changes in Plants 2.0

**DOI:** 10.3390/ijms24021773

**Published:** 2023-01-16

**Authors:** Tomasz Hura, Katarzyna Hura, Agnieszka Ostrowska

**Affiliations:** 1 Polish Academy of Sciences, The Franciszek Górski Institute of Plant Physiology, Niezapominajek 21, 30-239 Kraków, Poland; 2 Department of Plant Breeding, Physiology and Seed Science, Faculty of Agriculture and Economics, Agricultural University, ul. Podłużna 3, 30-239 Kraków, Poland

Plant adaptation to soil drought is a topic that is currently under investigation. Genetic and molecular studies have identified genes and proteins involved in the mechanisms of adaptation to environmental stresses. Climate change, manifested, e.g., by long-lasting soil droughts, render this type of research very important and necessary. Studies based on whole-genome sequencing techniques are particularly relevant and promising, as they enable us to determine the precise chromosome location of genes in economically important crops. From a long-term perspective, the mapping of drought resistance genes on the chromosomes may help growers to develop resistant crops and sustain crop production [1,2].

Six interesting research papers included in the second volume of the Special Issue, entitled “Drought-Stress Induced Physiological and Molecular Changes in Plants 2.0”, were compiled to present the latest findings on the molecular basis of plant adaptation to drought stress [2]. This Special Issue introduces the results of studies based on various experimental models (epiphytic orchid, crops, legumes, fruit tree) and covers genetic, molecular, biochemical, and physiological aspects.

The study conducted by Han et al. [3] reports the genome-wide identification of the *AP2/ERF* (*APETALA2/Ethylene Responsive Factor*) gene family, associated with abiotic stress in *Dendrobium catenatum* (*Dc*). A total of 120 identified *DcAP2/ERF* genes were subjected to a comprehensive phylogenetic analysis and were scrutinized for their structure, domain visualization, and promoter motif prediction. The paper also describes the expression patterns of the *AP2/ERF* family in different tissues and under low-temperature stress. The authors used qRT-PCR (quantitative Real-Time PCR) to detect twelve *DcAP2/ERF* genes expressed under drought stress and then analyzed the properties and functions of *DcAP2ERF#96*. They found that the heterologous expression of *DcAP2ERF#96* inhibits nine ABA (abscisic acid) downstream genes, including *P5CS1* and *RD29A*. The activities of these two genes are often investigated to determine the intensity of abiotic stresses. Moreover, in *D. catenatum*, the authors identified the DREB2A (Dehydration-Responsive Element Binding 2A) protein that interacts with *DcAP2ERF#96*, demonstrated the significant role of the *AP2/ERF* family, and highlighted a few candidate genes related to abiotic stress.

Li et al. [4] used proteomic analysis to reveal different mechanisms of response to PEG stress in drought-sensitive and drought-resistant sorghums. The work indicates that sorghum (*Sorghum bicolor* (L.) Moench) is the fifth most important grain crop in the world, grown mainly in arid and semi-arid tropical regions. A total of 102 differentially abundant proteins were obtained from drought-sensitive and drought-resistant varieties. The drought-sensitive varieties responded with the upregulation of proteins involved in the tyrosine metabolism pathway performing defense functions. The drought-resistant varieties showed enhanced sphingolipid biosynthesis, the promotion of the TCA (tricarboxylic acid) cycle, interference in triterpenoid metabolite synthesis, and changes in aminoacyl-tRNA biosynthesis. Additionally, 17 candidate proteins related to drought stress were verified by qRT-PCR. These results were concordant with those of the proteomic analysis. In conclusion, the authors emphasized the need for further research aiming to confirm the specific functions of the 17 key candidate sorghum proteins expressed under drought stress.

The article of Wei et al. [5] focuses on the genome-wide identification of the ERF transcription factor family and the functional analysis of the drought-stress-responsive genes in *Melilotus albus* (*Ma*). The authors identified a total of 100 *MaERF* genes containing a single AP2 domain sequence (*MaERF001* to *MaERF100*). Their bioinformatic analysis confirmed that segmental duplication may play a key role in the expansion of the *M. albus* ERF gene family. The analysis of *cis*-acting elements demonstrated that the *MaERF* genes may participate in various hormonal responses and abiotic stresses. A total of twenty *cis*-acting elements were counted. Moreover, four genes up-regulated under drought stress (*MaERF008*, *MaERF037*, *MaERF054*, and *MaERF058*) were overexpressed in yeast and enhanced the species’ tolerance to drought. This paper improves our understanding of the molecular mechanisms shaping the response of *M. albus* to drought. The authors also emphasize the need for further studies on the functions of the promising potential candidate genes identified in their study.

In another paper on *Melilotus albus*, Wang et al. [6] reported on the genome-wide identification of the GRAS family genes (derived from Gibberellin Insensitive—GAI, Gal Repressor—RGA and Scarecrow—SCR) and expression analysis of various tissues under different abiotic stresses. The GRAS gene family is a plant-specific family of transcription factors involved in the regulation of numerous metabolic pathways that are responsible, to name a few examples, for plant growth and development and responses to environmental stresses. The work identified 55 *MaGRAS* genes, which were grouped into eight subfamilies based on a phylogenetic analysis and found to be unevenly distributed on eight chromosomes. The analysis of the gene structure showed that *MaGRAS* genes have few introns and are highly conserved in *M. albus*. Moreover, their promoter region contains plant hormone response elements and stress response elements. Gene expression studies based on RNA-seq data and qRT-PCR confirmed the important role of the *MaGRAS* genes in plant growth and development and responses to abiotic stress or stress induced by abscisic acid treatment. The authors also demonstrated that the *MaGRAS* genes (*MaGRAS12*, *MaGRAS33*, and *MaGRAS34*) improve yeast’s tolerance to abiotic stresses.

Ao et al. [7] presented a genome-wide analysis and profile of the UDP-glycosyltransferases family in alfalfa (*Medicago sativa* L.) (*Ms*) under drought stress. The family of 1 UDP-glycosyltransferases (UGT) is involved, inter alia, in the regulation of plant growth and stress resistance. The analysis identified 409 UGT genes, and their phylogenetic tree, chromosomal location, duplication events, and exon-intron structures, conserved motifs, and *cis*-regulatory elements were evaluated (*MsUGT*). RNA-seq data and qRT-PCR confirmed the expression of *MsUGT* genes in various tissues under different abiotic stresses (drought, ABA treatment). Moreover, the heterologous expression of the *MsUGT* genes in yeasts indicated that *MsUGT003* and *MsUGT024* are involved in the response to drought stress and ABA signaling. The research reported in this paper may be crucial for exploring the function of *MsUGT* genes in drought stress and in the breeding of alfalfa. Nevertheless, in their conclusion, the authors emphasized the need for further studies to confirm the function of *MsUGT* genes.

The article by Liang et al. [8] focuses on the transcriptome and physiological analyses of a navel orange mutant (MT) with improved drought tolerance and water use efficiency (WUE) caused by the increased accumulation of cuticular wax and ROS (Reactive Oxygen Species)—scavenging capacity. The MT was found to produce greater total amounts of waxes and aliphatic wax compounds, including n-alkanes, n-primary alcohols, and n-aldehydes, than the wild-type (WT) plant. This resulted in a decreased cuticular permeability and, finally, improvements in the drought tolerance and WUE. In comparison with the WT, the MT showed lower contents of malondialdehyde and hydrogen peroxide, significantly higher levels of proline and soluble sugars, and an increased activity of superoxide dismutase, catalase, and peroxidase. These findings may indicate the improved drought stress tolerance capacity of the MT plants. Transcriptomic studies revealed the involvement of seven structural genes in wax transport and biosynthesis (*CsCER3-LIKE*, *CsABCG11-LIKE*, and *CsABCG21-LIKE*), MAPK (Mitogen-Activated Protein Kinases) cascade (*CsMEKK1-LIKE*), and ROS scavenging (*CsSOD1-LIKE*, *CsPRX5-LIKE*, and *CsPRX10-LIKE*). Seven other genes encoding transcription factors (*CsERF4-LIKE*, *CsERF9-LIKE*, *CsMYB62-LIKE*, *CsZAT10-LIKE1*, *CsZAT10-LIKE2*, *CsWRKY27-LIKE*, and *CsWRKY29-LIKE*) might play important roles in promoting cuticular wax accumulation and improving drought tolerance and WUE in the MT. The authors emphasized that their findings confirmed the important roles of cuticular waxes in plant adaptation to drought and provided information on various candidate genes that may play a role in improving drought stress tolerance.

In summary, the negative impacts of soil drought on crop growth and productivity and, consequently, on food production will become a major threats in the near future. This fact has sparked great interest in the identification of the key and effective mechanisms of plant adaptation to water deficit in the soil among researchers. Undoubtedly, the research papers published in this Special Issue enrich our knowledge of the molecular responses of plants to soil drought stress, providing essential and valuable information.

Finally, we would like to express our gratitude to the authors and reviewers for their invaluable work toward the publication of this Special Issue. We hope that the works published herein will inspire plant biology researchers to discover new research paths.
